# Establishment of a Predictive *In Vitro* Assay for Assessment of the Hepatotoxic Potential of Oligonucleotide Drugs

**DOI:** 10.1371/journal.pone.0159431

**Published:** 2016-07-21

**Authors:** Sabine Sewing, Franziska Boess, Annie Moisan, Cristina Bertinetti-Lapatki, Tanja Minz, Maj Hedtjaern, Yann Tessier, Franz Schuler, Thomas Singer, Adrian B. Roth

**Affiliations:** 1 Roche Pharmaceutical Research and Early Development, Pharmaceutical Sciences, Roche Innovation Center Basel, F. Hoffmann-La Roche Ltd, Basel, Switzerland; 2 Roche Pharmaceutical Research and Early Development, Therapeutic Modalities, Roche Innovation Center Copenhagen A/S, Hørsholm, Denmark; Jadavpur University, INDIA

## Abstract

Single stranded oligonucleotides (SSO) represent a novel therapeutic modality that opens new space to address previously undruggable targets. In spite of their proven efficacy, the development of promising SSO drug candidates has been limited by reported cases of SSO-associated hepatotoxicity. The mechanisms of SSO induced liver toxicity are poorly understood, and up to now no preclinical *in vitro* model has been established that allows prediction of the hepatotoxicity risk of a given SSO. Therefore, preclinical assessment of hepatic liability currently relies on rodent studies that require large cohorts of animals and lengthy protocols. Here, we describe the establishment and validation of an *in vitro* assay using primary hepatocytes that recapitulates the hepatotoxic profile of SSOs previously observed in rodents. *In vitro* cytotoxicity upon unassisted delivery was measured as an increase in extracellular lactate dehydrogenase (LDH) levels and concomitant reduction in intracellular glutathione and ATP levels after 3 days of treatment. Furthermore, toxic, but not safe, SSOs led to an increase in miR-122 in cell culture supernatants after 2 days of exposure, revealing the potential use of miR122 as a selective translational biomarker for detection of SSO-induced hepatotoxicity. Overall, we have developed and validated for the first time a robust *in vitro* screening assay for SSO liver safety profiling which allows rapid prioritization of candidate molecules early on in development.

## Introduction

Single stranded oligonucleotides (SSOs) can bind by Watson-Crick hybridization to an RNA target. This binding recruits RNAseH, which induces cleavage and degradation of the target RNA. This mechanism can be applied to specifically alter disease-relevant protein expression. Thus, SSOs are an attractive drug modality that gained increasing interest over the past years. However, despite three decades of SSO drug development, the clinical success has been very limited and led to only two FDA approved drugs [1]. Successes have been hampered initially by insufficient SSO stability and delivery to target tissue. Drug-induced toxicities including hepatotoxicity and pro-inflammatory effects further complicated drug development [1].

Although these toxicities occur in a concentration-dependent manner, there are SSOs which highly accumulate in the liver without causing harm to cells, while other molecules accumulate to a lesser extent, but exert significant hepatotoxicity. The mechanisms of these effects are poorly understood and may be caused by 1) hybridization of the respective SSO to non-intended RNA targets due to sequence similarity (hybridization-dependent off-target toxicity [2, 3]) or 2) binding of the SSO to intracellular proteins, thereby interfering with their function and ultimately leading to toxicity (hybridization independent toxicity [4, 5]).

The major adverse effects caused by SSOs *in vivo* can be summarized as accumulation and toxicity in liver and kidney as well as inflammatory reactions at the site of injection and flu like-symptoms [5]. Preclinically, whole body autoradiography of SSO-treated mice revealed that the main organs of SSO exposure after i.v. application are liver, kidney and spleen [6, 7], which are also the main organs where SSO-related toxicities are most often observed. SSO-associated liver and renal toxicities as well as immunotoxicity have been assessed early on in development using mainly rodent *in vivo* tests. Mice and rats treated with toxic SSOs showed significantly increased plasma aminotransferase levels (ALT and AST) and histopathological evidence for necrosis and apoptosis in liver and kidney [8, 9]. Furthermore, toxic SSOs induced lymphoid hyperplasia and organ cell infiltrates in rodents [10]. Early rodent toxicity tests performed to address SSO safety liabilities come with a high attrition rate and require large sets of animal cohorts for screening purposes which pose a significant challenge for SSO development from an ethical, cost, resource and time point of view. Recently it has been shown that certain sequence motifs are associated with hepatotoxicity risk [8, 11], but until now, no *in vitro* test has been identified that is able to recapitulate and predict the *in vivo* effect in cellular models [12] and thus is suited to replace *in vivo* testing.

To address this limitation, we have developed an *in vitro* screening assay to profile SSOs for liver liability before any animal studies are initiated. We successfully recapitulated the *in vivo* hepatotoxic profile by 1) testing a panel of SSOs of known *in vivo* liability, 2) using species-relevant primary hepatocytes, 3) applying SSOs to hepatocytes without the assistance of delivery technology and 4) selecting readouts with *in vivo* comparators. Remarkably, these experimental conditions successfully recapitulated the *in vivo* hepatotoxic profile of the tested SSOs. In addition to a decrease in cell viability, we report elevated levels of IL1α, MIP1α and miR-122 in the supernatant of primary hepatocytes exposed to toxic, but not safe SSOs. The assay presented here allows for the first time a rapid, early *in vitro* assessment of a key liability of SSOs. It can be used to prioritize lead series before initiation of animal studies and has the potential to significantly reduce the hepatotoxicity risk during SSO development.

## Materials and Methods

### Mouse liver perfusion and hepatocyte and non-parenchymal cell isolation

All procedures were conducted in strict adherence to the Swiss federal ordinance on animal protection and welfare, according to the rules of the Association for Assessment and Accreditation of Laboratory Animal Care International (AAALAC), and with the explicit approval of the local veterinary authority (Kantonales Veterinäramt Basel-Stadt, Switzerland).

Primary mouse hepatocytes were isolated from 10- to 13-week old male C57Bl6 mice by a retrograde two-step collagenase liver perfusion. Briefly, fed mice were euthanized with sodium pentobarbital (120 mg/kg, i.p.). Perfusion tubing was inserted via the right ventricle into the *v*. *cava caudalis*. Following ligation of the *v*. *cava caudalis* distal to the *v*. *iliaca communis*, the portal vein was cut and the two-step liver perfusion was started. The liver was first perfused for 5 min with a pre-perfusing solution consisting of calcium-free, EGTA (0.5 mM)-supplemented, HEPES (20 mM)-buffered Hank's balanced salt solution, followed by a 12-min perfusion with NaHCO3 (25 mM)-supplemented Hank's solution containing CaCl2 (5 mM) and collagenase (0.2 U/ml; Collagenase Type II, Worthington). Flow rate was maintained at 7 ml/min and all solutions were kept at 37°C. After in situ perfusion, the liver was excised, the liver capsule was mechanically opened, the cells were suspended in William’s Medium E (WME) without phenol red (Sigma W-1878), and filtered through a set of nylon cell straines (40- and 70-mesh). Dead cells were removed by a Percoll (Sigma P-4937) centrifugation step (percoll density: 1.06 g/ml, 50g, 10 min) and an additional centrifugation in WME (50xg, 3 min).

The supernatant from the hepatocytes sedimentation step was kept for the isolation of the non-parenchymal cell (NPC) fraction. Cells were pelleted by a centrifugation step (1200rpm, 10min, Eppendorf 5810R) and resuspended in 12 mL William’s Medium E (WME) without phenol red (Sigma W-1878). NPCs were isolated by a Percoll gradient (23% (upper phase) and 50% (bottom phase); Sigma P-4937) centrifugation step (Percoll density: 1.06 g/ml, 1350g, 10 min, brake off). Cells were washed once in WME, centrifuged (200g, 5 min, Eppendorf 5810R) and resuspended in 13mL William’s Medium E (WME) containing 10% fetal calf serum, penicillin (100 U/ml), streptomycin (0.1 mg/ml).

### Cell- culturing and oligonucleotide exposure

Freshly isolated primary mouse or cryopreserved human (BioreclamationIVT, Brussels, Belgium) hepatocytes were suspended in WME supplemented with 10% fetal calf serum, penicillin (100 U/ml), streptomycin (0.1 mg/ml) at a density of approx. 5 x 10^6^ cells/ml and seeded into collagen-coated 96-well plates (Becton Dickinson AG, Allschwil, Switzerland) at a density of 0.25 x 10^5^ cells/well (mouse) or 0.4 x 10^5^ (human). Cells were pre-cultured for 3 to 4h allowing for attachment to cell culture plates before start of treatment with oligonucleotides. Seeding medium was replaced by 90 μl of serum free WME and 10 μl of oligonucleotide stock solutions in PBS were added to the cell culture and left on the cells for 3 days.

Mouse NPCs were plated into collagen-coated 96-well plates (Becton Dickinson AG, Allschwil, Switzerland) at a density of approx. 0.25 x 10^5^ cells/well. For co-cultures primary mouse hepatocytes, suspended in WME supplemented with 10% fetal calf serum, penicillin (100 U/ml), streptomycin (0.1 mg/ml) at a density of 0.25 x 10^5^ cells/well, were seeded after 30 min on top of the NPC layer. Cells were cultured for 3 to 4h allowing for attachment to cell culture plates before start of treatment with oligonucleotides as described above.

HepG2 cells were cultivated at app. 80% confluence in MEM medium with GlutaMax (Gibco#41090), supplemented with 10% fetal calf serum. Cells were detached with Accutase (Sigma #A6964) and seeded into collagen-coated 96-well plates (Becton Dickinson AG, Allschwil, Switzerland) at a density of 0.5 x 10^5^ cells/well. Cells were pre-cultured at 37°C and 5%CO2 overnight, allowing for attachment before start of treatment with oligonucleotides described above.

### RNA isolation and qPCR

mRNA purification from mouse hepatocytes was performed using the RNeasy 96 Kit (Qiagen, Hombrechtikon, Switzerland) including an RNAse free DNAse I treatment according to the manufacturer’s instructions. cDNA was synthesized using iScript single strand cDNA Synthesis Kit (Bio-Rad Laboratories AG, Reinach, Switzerland). Quantitative real-time PCR assays (qRT-PCR) were performed using the Roche SYBR Green I PCR Kit and the Light Cycler 480 (Roche Diagnostics, Rotkreuz, Switzerland) with specific DNA primers. Analysis was done by the ΔΔCt threshold method to determine expression relative to RPS12 mRNA. Each analysis reaction was performed in duplicate, with two samples per condition.

### LDH, Albumin, GSH and ATP assays

Lactate dehydrogenase (LDH) released into the culture media was determined using a Cytotoxicity Detection Kit (Roche 11644793001, Roche Diagnostics GmbH Roche Applied Science Mannheim, Germany) according to the manufacturer's protocol. Albumin secretion was quantitated using mouse albumin ELISA Kit from Alpco diagnostics (41-ALBMS-E01). Intracellular GSH levels were determined in intact cells by a fluorescent assay using monochlorobimane (Fluka, 69899). In brief after removal of the cell supernatant, 100 μl of Krebs Henseleit Buffer (KHB) was added to the wells and background fluorescence was recorded. Next 50 μl of a 300 μM solution of monochlorobimane dissolved in KHB was added to the cells and incubated for 15 min at 37°C, before the reaction was stopped by removal of the monochlorobimane solution and addition of 100μL of fresh KHB to each well. Fluorescence was measured using a VictorV3 reader. For the determination of cellular ATP levels the CellTiter-Glo^®^ Luminescent Cell Viability Assay (G9242, Promega Corporation, Madison WI, USA) was used according to the manufacturer's protocol.

All Experiments were performed in triplicates.

### Apoptosis assay

Caspase-3/7 activity was determined using the Caspase-Glo^®^ 3/7 Assay (Promega Corporation, Madison WI, USA). In brief, Caspase-Glo^®^ 3/7 reagent was added to the cells at indicated time points, incubated for 30 min, before luminescence was determined on an Enspire multi-mode plate reader (Perkin Elmer) according to the manufacturer's instructions.

### Cytokine measurement

25 μl of supernatants from hepatocyte/NPC co-cultures were collected after 24, 48 and 64 hours of LNA treatment and stored at -20°C until analysis. For cytokine profiling, supernatants were thawed on ice, diluted 2x in sample dilution buffer (BioRad catalog # M60-009RDPD) and analyzed by multiplex ELISA using a mouse cytokine 23-plex (Bio-Plex Pro Mouse Cytokine 23-plex Assay, catalog # M60009RDPD) and Bio-Plex^®^ 200 Systems (BioRad) according to the manufacturer’s instructions. The 23 analytes were: IL-1α, IL-1β, IL-2, IL-3, IL-4, IL-5, IL-6, IL-9, IL-10, IL-12 (p40), IL-12 (p70), IL-13, IL-17A, Eotaxin, G-CSF, GM-CSF, IFN-γ, KC, MCP-1, MIP-1α, MIP-1β, RANTES and TNF-α. Data of selected analytes are reported as average concentrations and standard deviations (SD) of triplicate wells.

### miRNA analysis

To isolate miRNAs, 70 ul of cell culture supernatants (SN) were collected and the RNA was isolated using the miRNeasy Mini Kit (Qiagen, Germany, Catalog number 217004) following the manufacturer’s instructions. A volume of 1 μl of RNA was used for the cDNA synthesis using the cDNA TaqMan^®^ MicroRNA Reverse Transcription kit (Applied Biosystems, Thermo Fischer Scientific, USA, (Catalog number 4366596) and the expression of miR-122 was determined using a TaqMan miRNA assay (hsa-miR-122-5p; Applied Biosystems, Thermo Fischer Scientific, USA). Briefly, each sample was analyzed in duplicates and the reactions were performed using 1.33 μL of cDNA in a final volume of 20 μL with TaqMan^®^ Fast Advanced Master Mix reagents (Applied Biosystems, Thermo Fischer Scientific, USA). The PCR conditions were standardized to 95°C for 20 s followed by 40 cycles of 95°C for 1 s and 60°C for 20 s. The reactions were carried out on a QuantStudio 12K Flex Real-Time PCR System (Applied Biosystems, Lyfe technologies, USA), the EDS files were loaded in to the Quantstudio 12K Flex Software v1.2.2 and the raw Ct values were calculated using automatic baseline and fixed threshold values. The fold changes in microRNA expression were calculated according to the ΔΔCt-method, using spiked-in ath-miR-159 for normalization [13]. Each analysis reaction was performed in duplicate, with two samples per condition.

## Results

**Fig 1:**
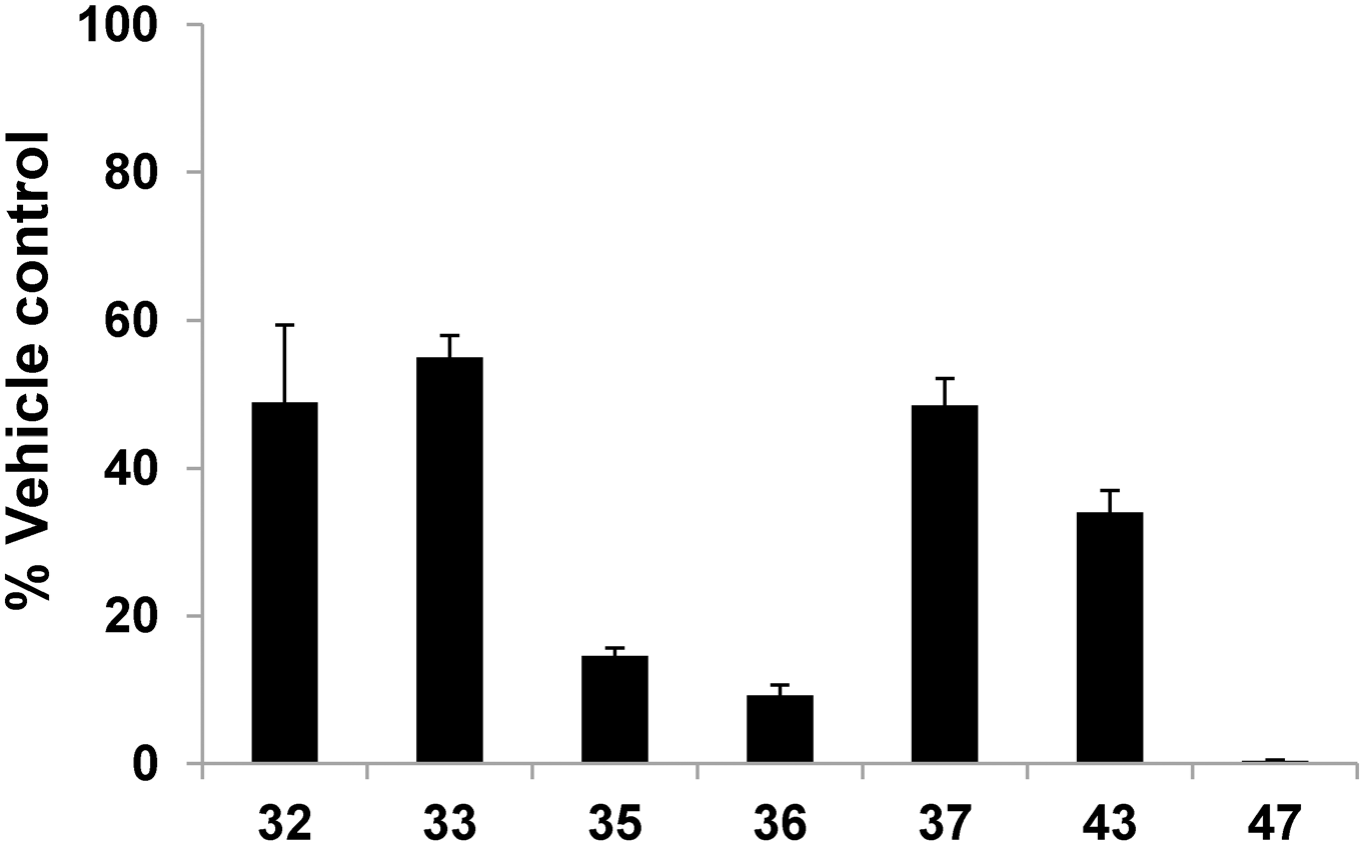
SSO induced target knock down in primary mouse hepatocytes. Normalized expression of myd88 mRNA after 48 hour treatment of mouse hepatocytes with 30 μM of the respective oligonucleotide. Data are means ± SD.

For evaluation and validation of potential predictive *in vitro* hepatotoxicity assays, a set of SSOs with known mouse *in vivo* hepatotoxicity was selected. These SSOs had LNA-modified nucleotides in the wings (LNA gapmers) and were directed against mouse myd88 mRNA (Table 1). Oligonucleotides were tested in freshly isolated primary mouse hepatocytes for 2–3 days by gymnotic delivery (without any carrier or transfection reagent; [14, 15]. In order to determine whether the oligonucleotides were taken up by the cells via the productive pathway, target knockdown after 48 hours of treatment was determined. Reduction of myd88 mRNA by 50–90% was observed in primary hepatocytes treated with SSOs 32, 33, 35, 36, 37, 43 and 47 indicating target engagement (Fig 1).

**Table 1:** Selection of tool SSOs with documented *in vivo* hepatotoxicity (ALT levels after 2 week treatment in mice with 5 x 15 mg/kg tail vein injection) that were used for the validation of *in vitro* hepatotoxicity assays.

SSO	ALT [U/L]	Sequence
**32**	64	5'- ^m^C_S_ A_S_ A_S_ a_S_ g_S_ g_S_ a_S_ a_S_ a_S_ c_S_ a_S_ c_S_ a_S_^m^C_S_ A_S_ T -3'
**33**	59	5'- ^m^C_S_ A_S_ A_S_ a_S_ t_S_ g_S_ c_S_ t_S_ g_S_ a_S_ a_S_ a_S_ c_S_ T_S_ A_S_ T -3'
**35**	67	5'- ^m^C_S_ T_S_^m^C_S_ a_S_ a_S_ c_S_ a_S_ t_S_ c_S_ a_S_ a_S_ g_S_ c_S_ A_S_ G_S_ T -3'
**36**	1889	5'- A_S_^m^C_S_ T_S_ g_S_ c_S_ t_S_ t_S_ t_S_ c_S_ c_S_ a_S_ c_S_ t_S_^m^C_S_ T_S_ G -3'
**37**	2368	5'- G_S_^m^C_S_^m^C_S_ t_S_ c_S_ c_S_ c_S_ a_S_ g_S_ t_S_ t_S_ c_S_ c_S_ T_S_ T_S_ T -3'
**43**	1890	5'- G_S_ A_S_ T_S_ g_S_ c_S_ c_S_ t_S_ c_S_ c_S_ c_S_ a_S_ G_S_ T_S_ T -3'
**47**	ND	5'- G_S_ A_S_ c_S_ a_S_ t_S_ t_S_ g_S_ c_S_ c_S_ t_S_^m^C_S_ T_S_ A -3'

ND: no ALT levels were determined, since group had to be sacrificed early due to severe toxicity. Small letters: nucleotides; s: phosphorothioate backbone; Capital letters: beta-oxy LNA, ^m^C: methylated cytosine.

In order to see, if the *in vivo* hepatotoxicity phenotype of our selected set of tool compounds can be recapitulated on an *in vitro* level, we evaluated various parameters in primary mouse hepatocytes treated with SSOs at different time points. Our readouts involved determination of hepatocyte function by measurement of albumin secretion, assessment of cell viability (ATP and LDH), oxidative stress (GSH), apoptosis induction (Caspase 3/7) and finally the secretion of potential biomarkers (miR122) and the influence of co-culturing non parenchymal liver cells as well as their influence on cytokine secretion.

**Fig 2:**
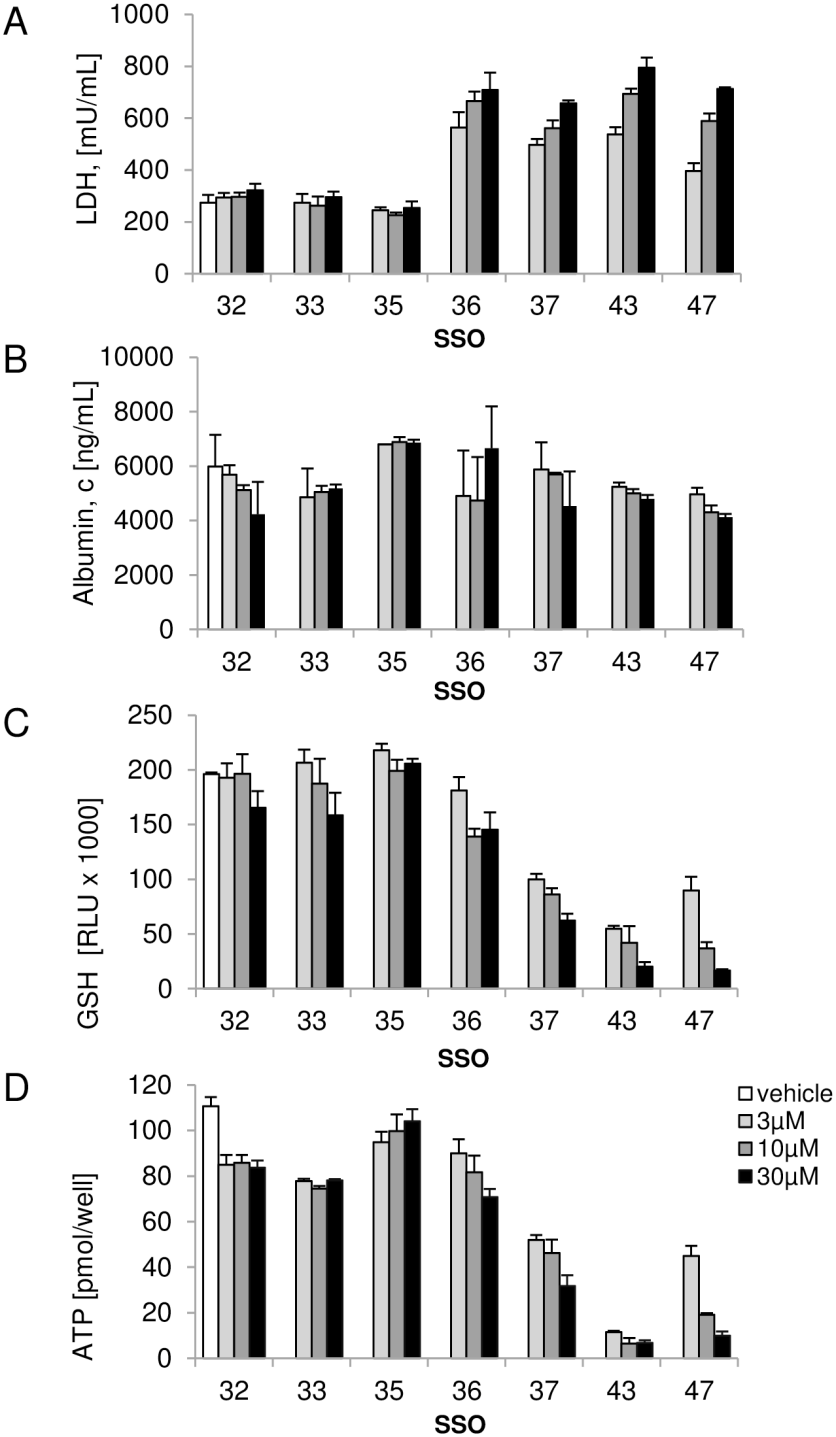
SSO induced toxicity in primary mouse hepatocytes. Secreted (A) LDH and (B) albumin levels and intracellular (C) GSH and (D) ATP concentrations after 3 day treatment with a tool set of hepatotoxic and non-hepatotoxic SSOs. Data are means ± SD.

The effects of SSOs on hepatocyte function and cell viability were first investigated *in vitro* by determination of albumin secretion and LDH levels in the supernatant as well as intracellular GSH levels and cellular ATP content. No significant changes in hepatocyte function or viability was observed 2 days after incubation of cells with all oligonucleotides (data not shown). After 3 days of treatment, LDH levels were significantly increased in supernatants of cells treated with toxic SSOs 36, 37, 43 and 47, while no change in LDH levels was seen with safe oligonucleotides 32, 33 and 35 (Fig 2A). In contrast, albumin secretion was not notably changed compared to vehicle treated hepatocytes for all SSOs tested indicating that albumin is not a sensitive marker for oligonucleotide induced toxicity in this setting (Fig 2B). In order to assess the potential of SSOs to induce oxidative stress, intracellular GSH content was determined after 3 days of exposure. Hepatotoxic SSOs 37, 43 and 47 induced a significant reduction in glutathione levels in mouse hepatocyte cultures while SSO 36 showed a mild but statistically highly significant reduction in GSH at 10 and 30 μM (p<0.001 using two-way ANOVA followed by Dunett’s multiple comparison test). Safe SSOs 32, 33 and 35 did not change GSH levels compared to vehicle (Fig 2C). A similar picture was obtained when assessing intracellular ATP levels which revealed a good differentiation between toxic and safe SSOs. Cellular ATP levels were slightly reduced in hepatocytes treated with safe SSOs 32, 33 and 35 compared to the vehicle group, but this effect was small and not concentration-dependent. Treatment with hepatotoxic SSOs, 37, 43 and 47 led to a marked reduction in ATP while SSO 36 showed a concentration dependent mild reduction in intracellular ATP concentration, which is at 30 μM statistical significant compared to the safe SSO 32 (Fig 2D).

When safe and toxic SSOs where tested in a non-primary cell model such as HepG2 using similar readouts, we did not see any of the above described effects (supporting information S1 Fig), underlining the importance of using the right cell model that is capable of gymnotic SSO uptake and trafficking similar to the physiological situation to recapitulate SSO induced toxicity.

**Fig 3:**
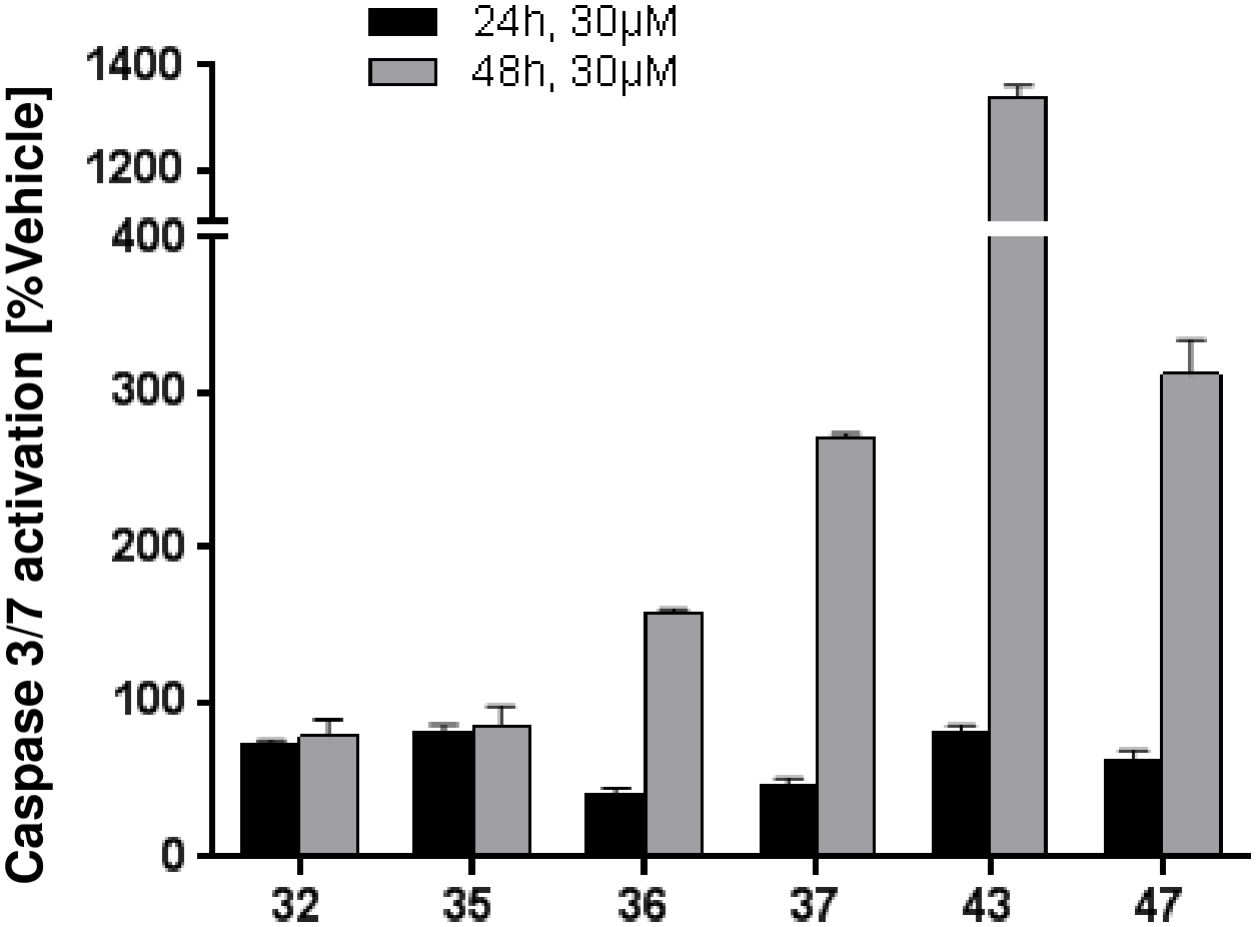
Apoptosis induced by SSOs in primary mouse hepatocytes. Changes in caspase-3/7 activation in primary mouse hepatocytes after 24 and 48 h of incubation with the respective SSO at 30 μM. Data are normalized to vehicle treated cells and are means ± SD.

In order to understand earlier events that ultimately result in reduction of cell viability after treatment with hepatotoxic SSOs, SSO-mediated effect on apoptosis by measuring caspase-3/7 activation at 3 different time points was assessed. After 24 hours of incubation, no activation of caspase-3/7 could be detected (Fig 3). At 48 hours, a clear increase in caspase-3/7 activation was observed with toxic SSOs 36, 37, 43 and 47 with the strongest response induced by SSO 43, while no effect was seen with safe SSOs 32 and 35. After 3 days of treatment increases in caspase-3/7 levels were still evident with toxic oligonucleotides (not shown). These signals perfectly correlated with the *in vitro* cytotoxicity profile of SSOs after 3 day treatment. These results clearly indicate that hepatotoxic SSOs induce apoptosis in mouse hepatocytes, which then finally leads to the changes in cell viability shown in Fig 2.

**Fig 4:**
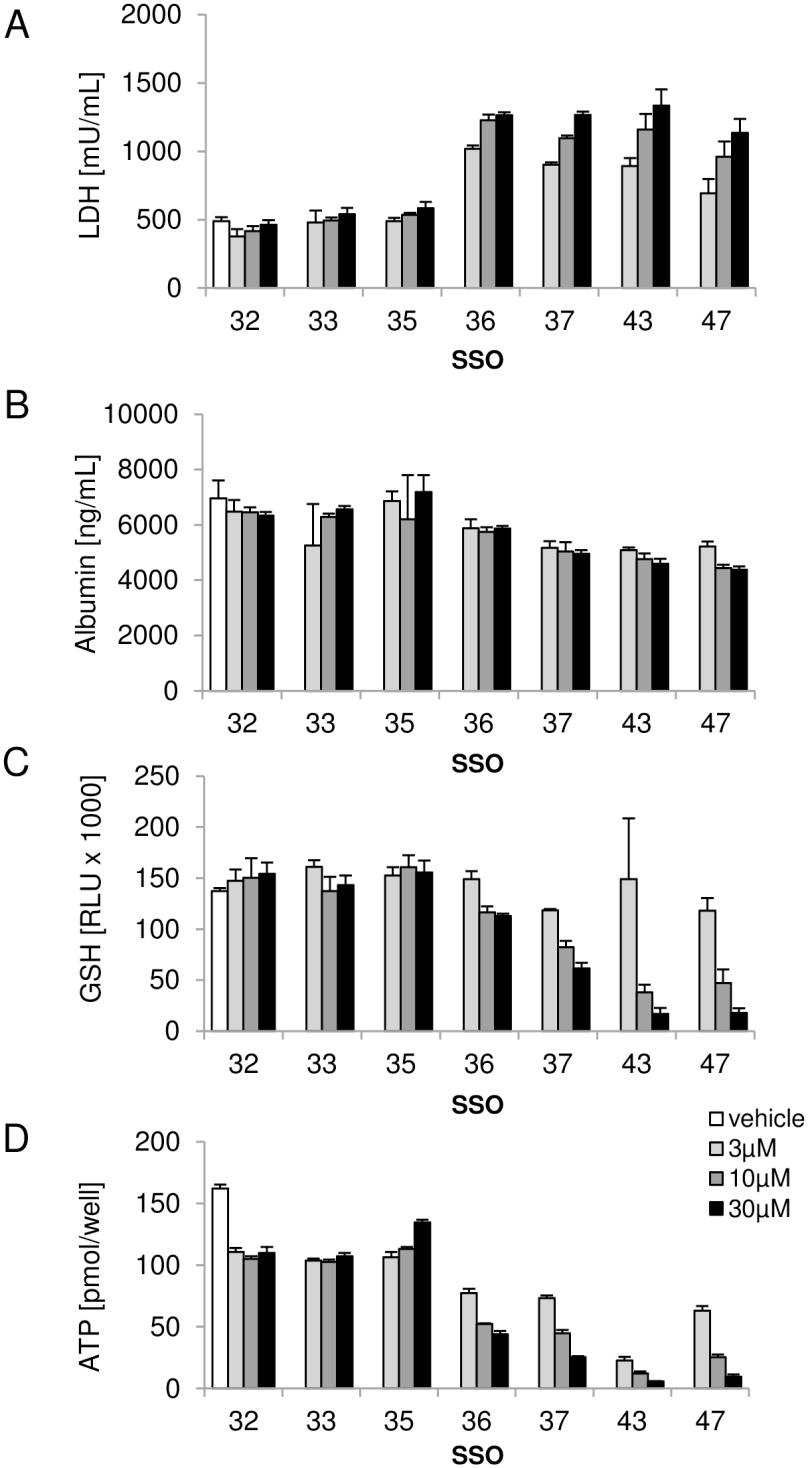
SSO induced toxicity in primary mouse hepatocyte NPC co-cultures. Secreted (A) LDH and (B) albumin levels and (C) intracellular GSH and (D) ATP concentrations after 3 day treatment of mouse primary hepatocyte NPC co-cultures with a tool set of hepatotoxic and non-hepatotoxic SSOs. Data are means ± SD.

It is well established that SSOs are taken up by non-parenchymal cells (NPC) in the liver [16] and NPCs are described to have a prominent contribution to SSO induced hepatotoxicity *in vivo* [5]. In order to determine the potential contribution of the non-parenchymal cell fraction to the degree of hepatotoxicity seen with SSOs, another set of *in vitro* experiments was conducted in mouse hepatocyte-NPC co-cultures. As shown in Fig 4, co-cultures treated with hepatotoxic SSOs 36, 37, 43 and 47 for 3 days showed increased LDH levels (A), slightly decreased albumin secretion (B), and a clear reduction in intracellular GSH levels (C) and ATP content (D) compared to vehicle treated co-cultures. None of these parameters changed after incubation with safe SSOs 32, 33 and 35. Compared to the results obtained with mouse hepatocyte monocultures, the co-cultures displayed similar sensitivity in LDH, GSH and ATP changes towards discrimination of toxic and non-toxic SSOs.

With a subset of 2 safe and 2 toxic SSOs (32, 35, 43 and 47) we evaluated, if the NPC fraction alone is functional and responding to SSO treatment (supporting information S2 Fig). In summary, we observed an increase in LDH levels in the supernatants of NPCs treated with toxic compounds 43 and 47, while no changes were seen with SSOs 32 and 35. These data demonstrate that both mouse primary hepatocytes and non-parenchymal cells are susceptible to SSO induced toxicity.

**Fig 5:**
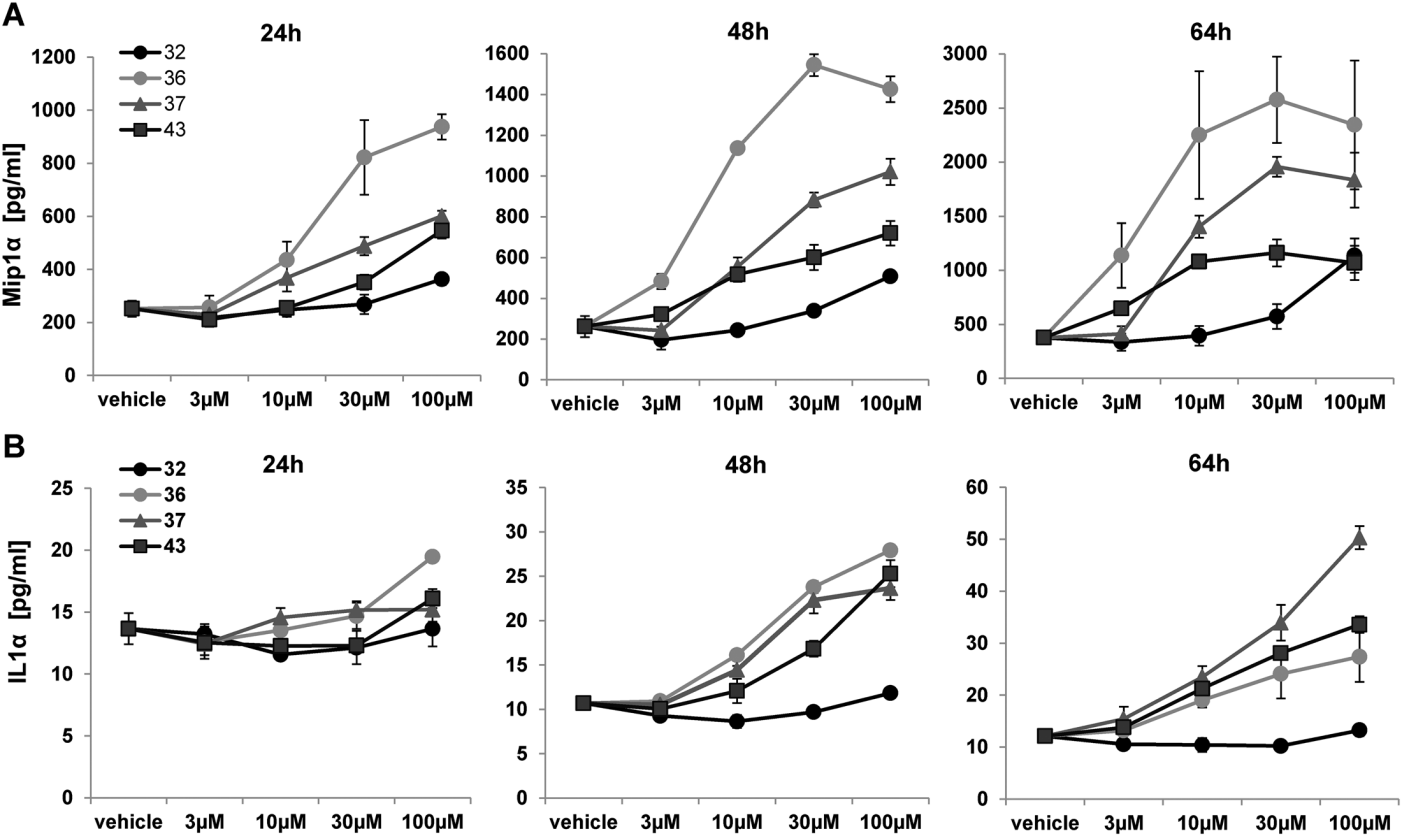
SSO induced cytokine secretion in primary mouse hepatocyte NPC co-cultures. Secretion of (A) MIP1α and (B) IL1α after 24, 48 and 64 hour treatment of primary mouse hepatocyte NPC co-cultures with safe SSO 32 and hepatotoxic SSOs 36, 43 and 47. Data are means ± SD.

To further elucidate the effect of oligonucleotides on hepatocytes and non-parenchymal co-cultures, changes in secreted cytokines and miR-122, a hepatocyte-specific marker for tissue damage [17, 18] were investigated upon SSO treatment. The cytokine secretion profile of safe (32) and hepatotoxic (36, 37 and 43) SSOs was determined using a multiplex Bio-Plex^®^ assay after 1, 2 and 3 days of SSO treatment. Of the 23 tested analytes (see material and methods), Macrophage Inflammatory Protein 1 alpha (MIP1α) and Interleukin 1 alpha (IL1α) showed a time-dependent response in mouse co-cultures treated with hepatotoxic SSOs. As shown in Fig 5, MIP1α was already induced up to 5 fold after 24 hours of treatment with toxic SSOs 36, 37 and 43. We also observed an increase in IL1α with toxic SSO with a later onset than MIP1α, clearly separating toxic and non-toxic SSO at 48 and 64 hours.

**Fig 6:**
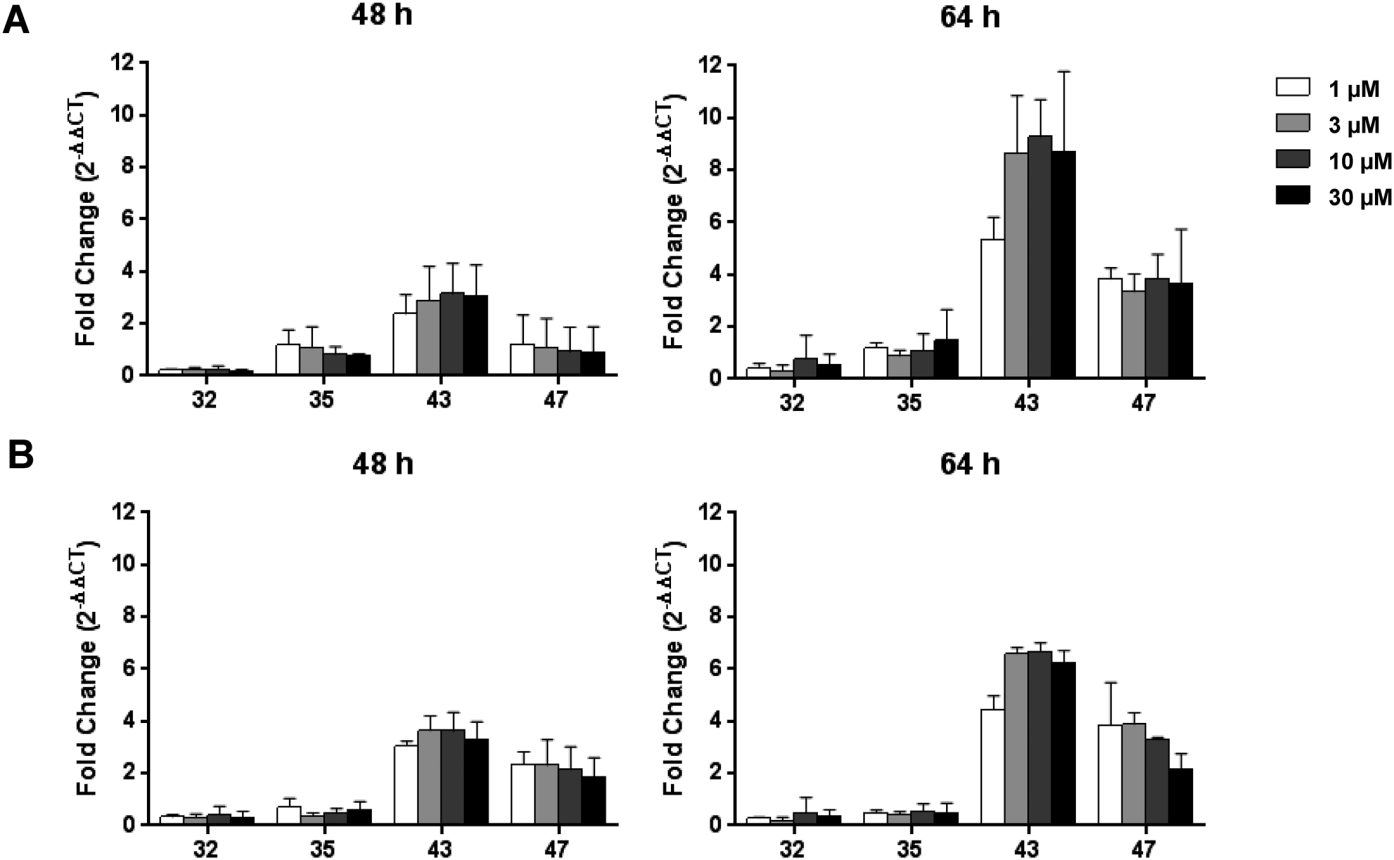
miR-122 release. (A) Mouse hepatocyte-NPC co-cultures or (B) hepatocyte monocultures were treated with the respective SSOs for 48 or 64 hours and cell-free supernatant was collected. The levels of miR-122 at 48 h and 64 h were assessed using real-time qPCR. miRNA levels were normalized to vehicle treated cells. Data are means ± SD.

miR-122 release into the supernatants was investigated in mouse hepatocyte monoculture and hepatocyte-NPC co-cultures treated with non-toxic SSO 32 and 35 and toxic SSOs 43 and 47. Increases in miR122 were observed already after 2 days of treatment with toxic SSOs (Fig 6). Although a 4-10-fold increase in miR122 was detected in the supernatants of co-cultures treated with SSOs 43 and 47 compared to vehicle (Fig 6A), the amount was not significantly higher than the miR-122 release observed when the hepatocytes were cultured alone (Fig 6B). Overall, these data further support the idea that liver injury observed *in vivo* is a direct result of SSO induced damage in each individual exposed liver cell type rather than an event confined to non-parenchymal cells only.

**Fig 7:**
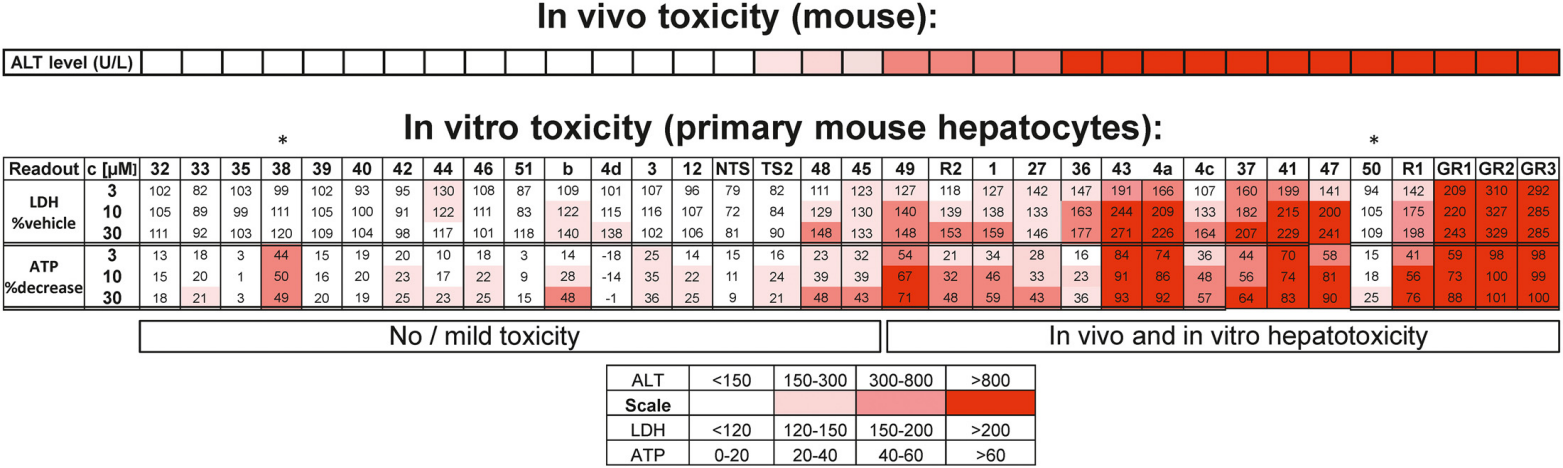
*In vitro* prediction of SSO induced hepatotoxicity in primary mouse hepatocytes. Increase in secreted LDH levels and reduction in cellular ATP levels after 3 day treatment of primary mouse hepatocytes with 34 SSOs with different *in vivo* hepatotoxicity as reported by plasma ALT concentration after sub-chronic treatment. *outliers.

To further validate this novel *in vitro* assay set up and to determine if the correlation of the *in vitro* toxicity pattern with mouse *in vivo* hepatotoxicity seen for our 7 model SSOs translates to a larger set of oligonucleotides, the tool set was extended to 34 SSOs targeting different mRNAs and showing a broad spectrum of mouse *in vivo* hepatotoxicity. Cellular ATP and secreted LDH were selected as readouts after 3 days of treatment in freshly isolated mouse hepatocyte monocultures, as these two assays turned out to be sufficient to recapitulate the *in vivo* toxicity pattern with our initial set of tool SSOs. As shown in Fig 7, there is good correlation for 32 out of 34 SSOs between *in vivo* hepatotoxicity and *in vitro* readouts. SSOs that induced increases in plasma ALT levels *in vivo* also clearly showed an increase in LDH and a reduction in ATP levels *in vitro* in mouse hepatocytes, with only 2 SSOs, where *in vivo* toxicity was not predicted with the cellular assay. Thus, this assay is suitable to predict the hepatotoxic potential of oligonucleotides prior to moving into first *in vivo* studies.

**Fig 8:**
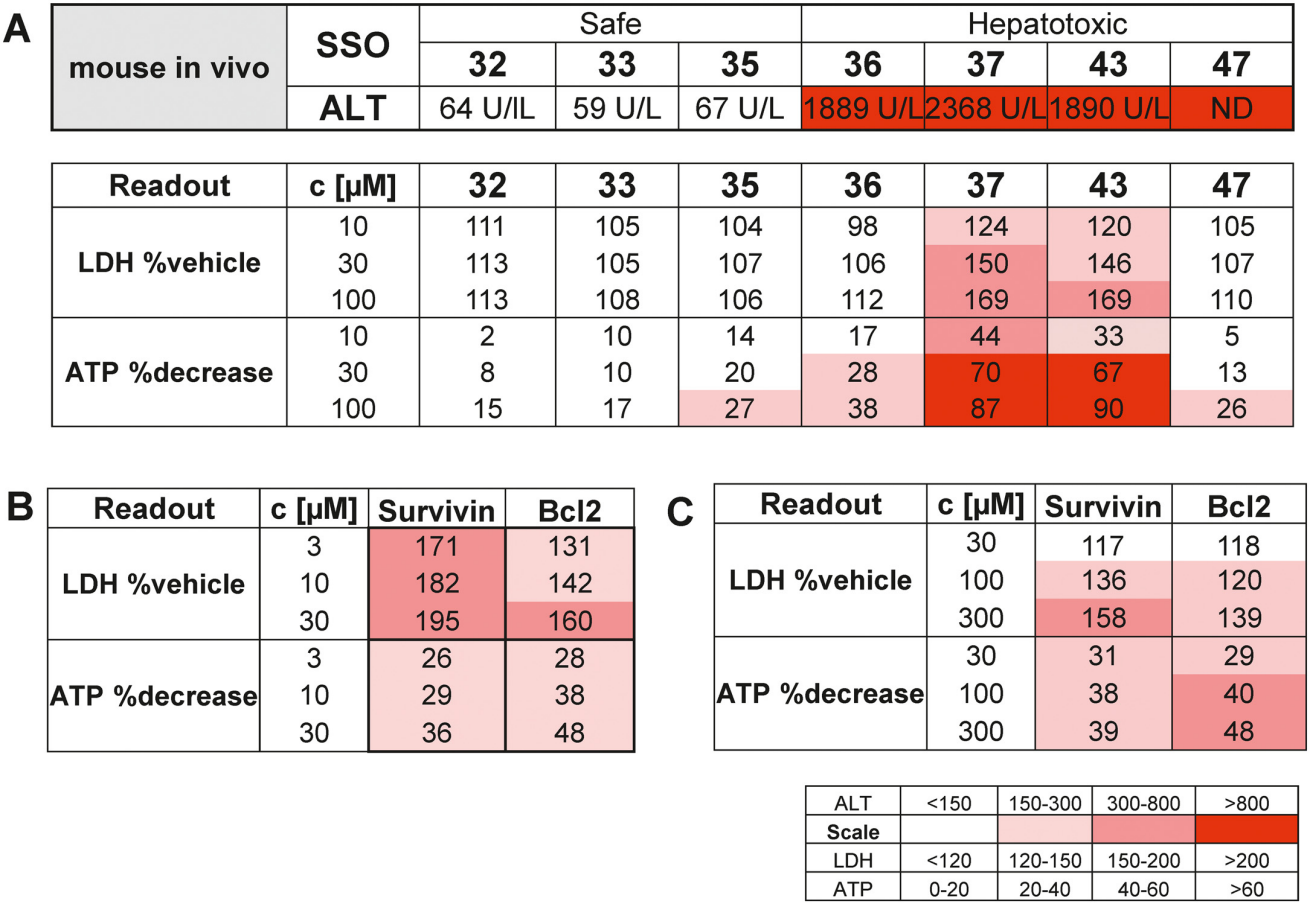
SSO induced toxicity in cryopreserved human hepatocytes. (A) Changes in secreted LDH and cellular ATP concentrations after 3 day incubation of human hepatocytes with a tool set of hepatotoxic and non-hepatotoxic myd88 SSOs and changes of ATP and LDH after 3 day treatment of (B) mouse hepatocytes and (C) human hepatocytes with two SSOs targeting Bcl2 and Survivin that have been tested in the clinic. Data are means from 2 experiments in triplicates.

Finally, in an attempt to establish a human relevant *in vitro* model, a similar protocol was established for human primary hepatocytes. Cytotoxicity readouts were measured 3 days after treatment with the myd88 tool SSOs, initially tested in primary mouse hepatocytes. A clear pattern reflecting *in vivo* innocuous versus *in vivo* hepatotoxic SSOs in human hepatocytes was obtained for 5 out of the 7 tested myd88 tool compounds in human hepatocytes (Fig 8A). Toxic SSOs 37 and 43 showed a dose dependent increase in LDH levels and decrease in intracellular ATP levels, whereas SSOs 32, 33 and 35 had no apparent effect. Lastly, in order to see the human relevance of this assay, two historical development SSOs targeting Survivin and Bcl2, with documented pre-clinical as well as clinical liver effects [19–21] were tested in mouse (Fig 8B) and human primary hepatocytes (Fig 8C). With both SSOs, increases in LDH levels and reduction in cellular ATP were observed in mouse and human cellular systems, suggesting reliability of the human hepatocyte assay for estimation of a potential clinical liver toxicity risk.

## Discussion

We here describe a novel *in vitro* assay which for the first time is able to capture the hepatotoxic potential of SSO drugs and can be used as a screen to prioritize molecules before *in vivo* studies are initiated. In order to allow for the detection of hybridization dependent off-target effects as well as aptameric non-hybridization dependent effects, SSOs were delivered to cells by gymnosis, without use of delivery vehicle or molecular conjugation. Using a balanced set of SSO reference molecules with documented *in vivo* liver toxicity, SSO-induced changes in LDH, GSH, ATP, caspase-3/7 activity, cytokine- and miR122-release were observed and correlated with the *in vivo* toxicity profile. Assessing this battery of cellular markers in a time- and concentration-dependent manner, a minimal set of required measurements could be defined, which allows to accurately detect hepatotoxic SSOs, namely assessment of LDH and ATP after 3 days of continuous treatment at 1–30 μM (Fig 2) in primary mouse hepatocytes. All seven selected tool SSOs used for assay development showed target reduction, and the extend of mRNA knockdown did not correlate with the toxic effect observed in the same cells or *in vivo* in mice, ruling out that some SSOs are not toxic in our setting just because of lack of exposure. Data from apoptosis induction experiments suggest a mechanism underlying SSO-induced toxicity involving early activation of this cellular process ultimately leading to cell death. Unlike earlier reports, where cellular caspase-3 activation was described in A549 or HepG2 cells transfected with SSOs, but no correlation between cellular toxicity and toxicities in mice could be observed [12], our *in vitro* caspase-3/7 activation data perfectly fit with *in vivo* the hepatotoxicity observed in mice.

As non-parenchymal cells have been shown to preferentially take up SSOs in the liver, it has been speculated that Kupffer cell activation and subsequent inflammatory processes may be initial events triggering hepatotoxicity [5, 16, 22]. In particular, in *in vivo* studies in mice with SSOs, an elevation of MIP1α and IL1α among other pro-inflammatory cytokines and chemokines in the liver has been reported [23]. We obtained similar results in our *in vitro* assay, when the effect of SSOs was investigated in hepatocyte NPC co-cultures. A significant increase in secreted MIP1α and IL1α levels was observed in co-cultures treated with toxic SSOs already after one or two days of treatment, respectively. However, when the cellular toxicity profile induced by our tool set of SSOs was compared in hepatocyte mono- and NPC co-cultures, no major difference in sensitivity towards detecting toxic SSOs *in vitro* was observed (Figs 2 and 4). These data, together with the fact that the established hepatocellular damage marker miR-122 was increased in both mono- and co-cultures (Fig 6) clearly suggest that in addition to an NPC-mediated component to acute hepatic toxicity of SSOs [5] there is a similar direct effect of SSOs on hepatocytes. The model established here not only serves to rapidly screen for bad actors early on in the pipeline, but could also be a model to increase the mechanistic understanding of SSO-induced hepatotoxicity which ultimately should lead to improved design of safe and efficacious drugs. A cornerstone of such a concept, which so far has been lacking in SSO drug development, is translation of pre-clinical findings to human [5]. A series of reports describe changes observed in rodent and non-rodent studies related to SSO-induced toxicity, but as the safest preclinical candidates progress into the clinic, the human relevance of the observed effects remained unclear so far. We therefore aimed to establish a human *in vitro* system for SSO-induced hepatotoxicity in parallel to the rodent one described, to compare data for our tool SSO set and explore, if a human cell system would be able to correctly predict molecules where the clinical liver profile is known (Fig 8). A good correlation between *in vitro* mouse and human hepatocyte toxicity was obtained for 5 out of 7 SSOs from our myd88 tool SSO set. Signal strength was generally weaker in human cells and required higher concentrations, but the relative ranking was the same as in the mouse system. If the difference observed between mouse and human hepatocytes for two out of the seven tool SSOs is a real species difference or just a matter of lower sensitivity cannot be answered yet due to the lack of respective clinical data and remains to be investigated. Importantly, the human hepatocyte model correctly reflected the human liver profile of two historical SSOs that showed grade 3 increases in liver enzymes in phase 1 clinical trials [19, 20].

Our results suggest that acute liver toxicity caused by SSOs can be triggered by a direct effect on hepatocytes, which seems reproducible across species albeit some differences were observed. Thus incorporation of rodent and human *in vitro* hepatotoxicity testing using primary hepatocytes will improve prioritization of SSO that will be moved into *in vivo* safety testing in rodents and higher species and addresses translation of effects to human.

In summary, we provide for the first time a reliable *in vitro* assay system which allows selection of SSO candidate drugs which have a lower risk for causing hepatotoxicity early on in development. The cellular system used here is based on primary cells which are able to take up SSO drugs in an unassisted manner thus representing a more *in vivo*-like model to study uptake, processing and potential hepatotoxicity of novel SSO drugs which is relevant for the *in vivo* situation.

## Supporting Information

S1 Fig SSO induced toxicity in HepG2 cells. Secreted LDH (upper panel) intracellular ATP (lower panel) concentrations after 3 day treatment with a tool set of safe (32, 35) and hepatotoxic (43, 47) SSOs. Data are means ± SD.
(TIF)

S2 Fig SSO induced cytotoxicity in primary mouse non-parenchymal cells. LDH levels in the supernatant of primary mouse NPCs after 2 day treatment with safe (32, 35) and toxic (43, 47) SSOs. Data are means ± SD.
(TIF)
