# Supplementary figures and images for: Establishment of a Predictive In Vitro Assay for Assessment of the Hepatotoxic Potential of Oligonucleotide Drugs
Source: PLoS One. 2016 Jul 21;11(7):e0159431. doi: 10.1371/journal.pone.0159431 (PMC4956313; doi:10.1371/journal.pone.0159431)

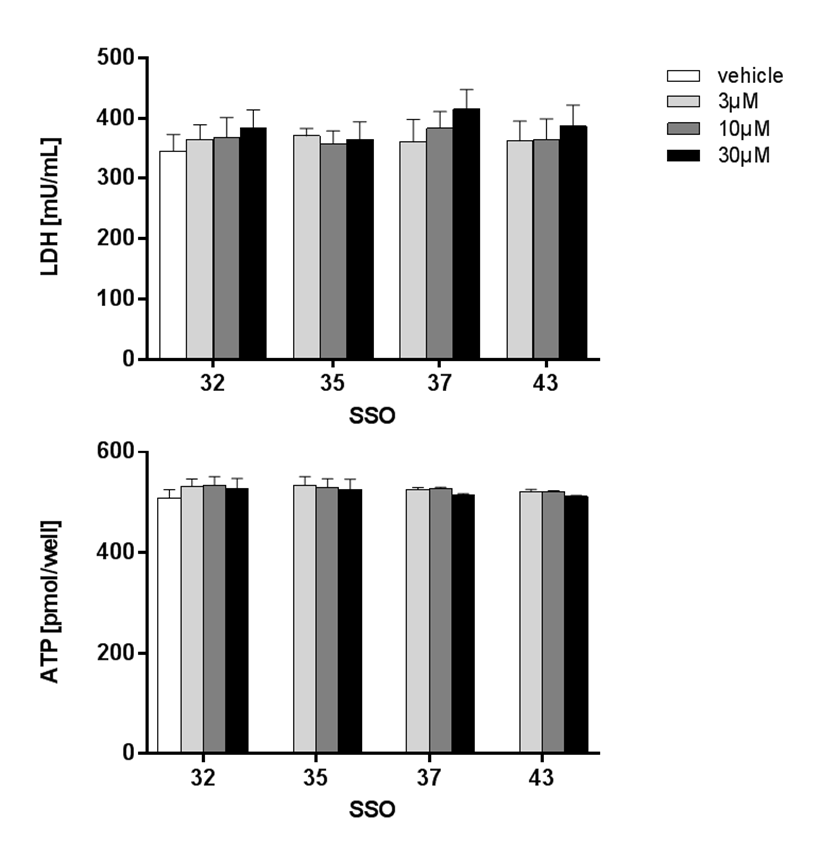

Supplement: S1 Fig — Secreted LDH (upper panel) intracellular ATP (lower panel) concentrations after 3 day treatment with a tool set of safe (32, 35) and hepatotoxic (43, 47) SSOs. Data are means ± SD. (TIF) [file pone.0159431.s001.tif]

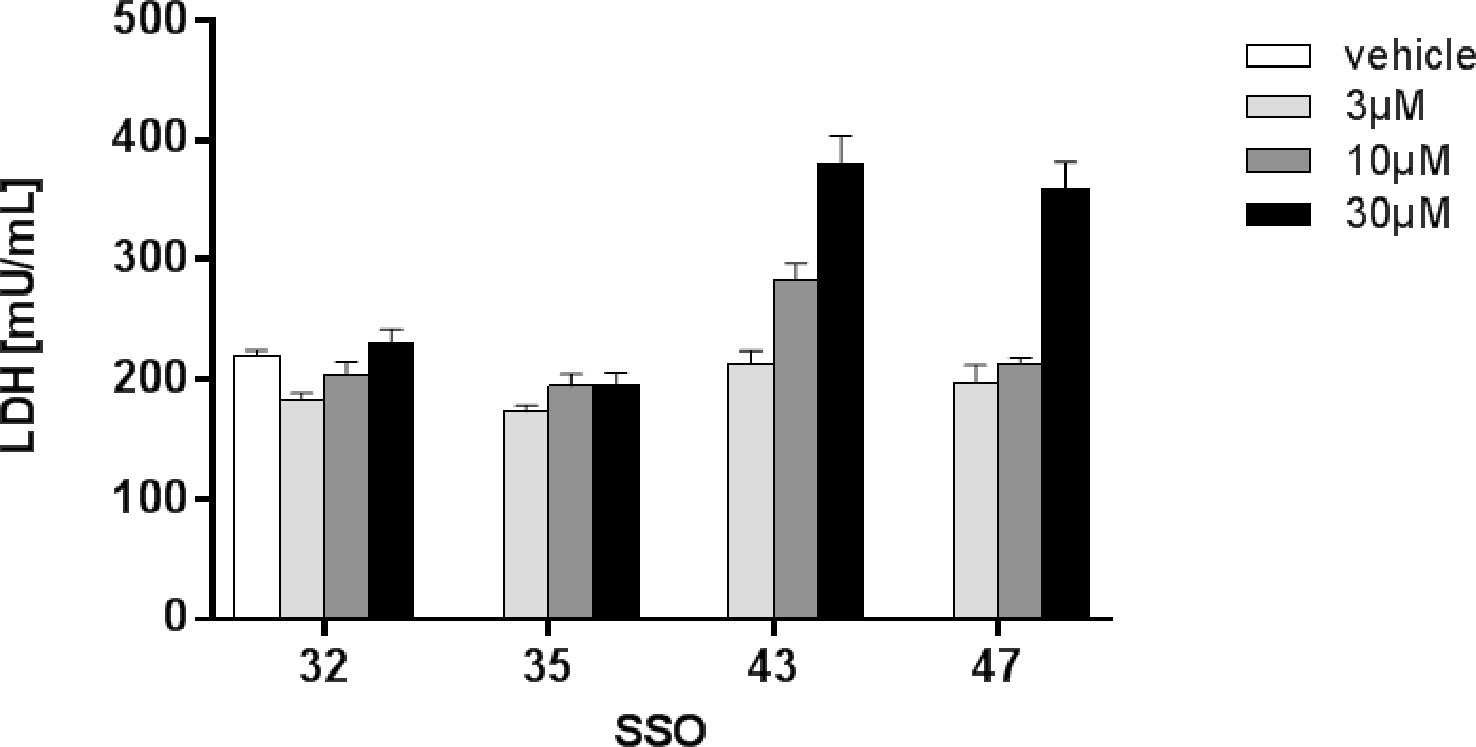

Supplement: S2 Fig — LDH levels in the supernatant of primary mouse NPCs after 2 day treatment with safe (32, 35) and toxic (43, 47) SSOs. Data are means ± SD. (TIF) [file pone.0159431.s002.tif]
